# *TBRG4* as a prognostic biomarker and key regulator of cell cycle and EMT in lung cancer

**DOI:** 10.17305/bb.2025.11353

**Published:** 2025-04-18

**Authors:** Ansheng Wang, Qiao Ge, Zhenkai Fan, Bing Xia, Zhao Jin, Haitao Liu, Haiwei Sang, Qicai Li, Congli Zhang, Haonan Zhu

**Affiliations:** 1 Department of Thoracic Surgery, The First Affiliated Hospital of Bengbu Medical University, Bengbu, Anhui, China; 2 Department of Respiratory and Critical Care Medicine, The First Affiliated Hospital of Bengbu Medical University, Bengbu, Anhui, China; 3 Department of Anesthesia, The First Affiliated Hospital of Bengbu Medical University, Bengbu, Anhui, China; 4 Department of Thoracic Surgery, Fuyang People’s Hospital, Fuyang, Anhui, China

**Keywords:** Lung cancer, TBRG4, cell cycle, epithelial-mesenchymal transition, prognostic biomarker

## Abstract

Transforming growth factor β regulator 4 (*TBRG4*) is upregulated in lung cancer, but its biological role and underlying mechanisms remain poorly understood. In this study, we analyzed pancancer gene expression profiles and clinical data from University of California, Santa Cruz Xena (UCSC Xena) to evaluate the prognostic significance of *TBRG4* using univariate and multivariate Cox regression analyses. Genes with a Pearson correlation coefficient above 0.4 with *TBRG4* in lung cancer were identified via UALCAN, followed by pathway enrichment analyses to explore their functional associations. To investigate *TBRG4*’s role in lung cancer progression, we assessed cell proliferation, colony formation, and cell cycle alterations in lung cancer cells following *TBRG4* knockdown. Western blot analysis was performed to examine the effects of *TBRG4* depletion on key cell cycle regulators and epithelial-mesenchymal transition (EMT) markers. Additionally, the biological significance of *TBRG4* was evaluated *in vivo* using a mouse xenograft model. *TBRG4* knockdown significantly inhibited cell proliferation and colony formation while inducing cell cycle arrest and apoptosis in lung cancer cells. Analysis of co-expressed genes in the The Cancer Genome Atlas - Lung Adenocarcinoma (TCGA-LUAD) cohort revealed enrichment in cell cycle-related pathways, aligning with our experimental findings. Furthermore, *TBRG4* depletion reduced EMT marker expression and suppressed tumor growth *in vivo*. Collectively, these findings suggest that *TBRG4* may serve as a promising prognostic biomarker and therapeutic target in lung cancer.

## Introduction

Lung cancer remains a leading cause of cancer-related deaths worldwide, accounting for approximately 20% of all cancer deaths in 2018 [1, 2]. Lung adenocarcinoma (LUAD), the predominant histological subtype, constitutes nearly half of all lung cancer cases [3]. Despite advancements in chemotherapy, targeted therapy, and immunotherapy [4], the prognosis for lung cancer remains poor. Approximately 70% of cases are diagnosed at advanced stages (III or IV), and the five-year survival rate remains below 20% [5]. Therefore, understanding the regulatory mechanisms of LUAD progression and identifying novel biomarkers for early diagnosis and treatment are critical. Transforming growth factor β regulator 4 (*TBRG4*), also known as CPR2 or FASTKD4, is encoded by the *TBRG4* gene on chromosome 7 [6]. *TBRG4* has been implicated in various diseases, including cancer [7, 8]. Previous studies have identified *TBRG4* as an oncogene in breast cancer, where its deficiency inhibits tumor cell migration and proliferation by promoting apoptosis [9]. Additionally, *TBRG4* has been associated with multiple myeloma, underscoring its potential role in tumorigenesis [10]. Our previous research identified significantly elevated TBRG4 protein levels in lung cancer tissues compared to normal tissues, suggesting its involvement in critical pathways such as cell cycle regulation. Building on these findings, the current study aims to systematically explore the role of *TBRG4* in LUAD by analyzing its mRNA expression using The Cancer Genome Atlas (TCGA) database. We further assess its prognostic significance across multiple lung cancer cohorts and investigate its biological functions through *in vitro* and *in vivo* experiments. By conducting co-expression analysis, pathway enrichment analysis, and validating results via western blotting, we aim to elucidate the molecular mechanisms through which *TBRG4* contributes to lung cancer progression. The significance of this study lies in identifying *TBRG4* as a novel biomarker and potential therapeutic target for LUAD. Understanding how *TBRG4* drives tumor growth and progression could pave the way for developing targeted therapies that improve early diagnosis and enhance treatment efficacy. This research holds promise for advancing precision medicine in lung cancer, with the potential to reduce mortality and improve patient outcomes.

## Materials and methods

### Bioinformatic statistics

We obtained pancancer gene expression profiles and clinical data from University of California, Santa Cruz Xena (UCSC Xena) (https://xenabrowser.net/datapages/) and normal tissue expression data from the GTEx dataset (https://www.gtexportal.org/home/). Normal tissues from GTEx were matched to corresponding TCGA cancer types after adjusting for batch effects. Duplicate samples from the same patient and those lacking sufficient clinical data were excluded. Statistical comparisons between two groups were conducted using Student’s *t*-test, while one-way ANOVA was used for comparisons involving more than two groups. A *P* value of < 0.05 was considered statistically significant. Univariate and multivariate Cox regression analyses were performed to evaluate whether TBRG4 functions as an independent prognostic factor, adjusting for clinical and pathological variables. Significant features from the univariate analysis were included in the multivariate Cox regression to determine their coefficients. Kaplan–Meier survival curves were used to compare survival outcomes between groups, and prognostic performance was assessed using receiver operating characteristic (ROC) curves. The prognostic value of TBRG4 was further validated in the GSE30129, GSE31210, CaArray, and GSE37745 cohorts using SurvExpress, a platform for cancer gene expression data linked to clinical outcomes. Genes with a Pearson correlation coefficient <0.4 with TBRG4 in LUAD were extracted from UALCAN. Functional annotation of these co-expressed genes was conducted using KEGG, HALLMARK, and Metascape analyses [11]. Additionally, gene set enrichment analysis (GSEA) was used to compare high- and low-risk patient subgroups, stratified based on the previously defined risk score.

### Cell culture

The human lung cancer cell lines (H1688, H1975, H1299, and A549) and normal human lung epithelial cells (BEAS-2B) were obtained from the Cell Bank of the Chinese Academy of Sciences (Shanghai, China). H1299, H1688, and H1975 cells were cultured in RPMI-1640 medium, A549 cells in Ham’s F-12K medium, and BEAS-2B cells in Dulbecco’s Modified Eagle Medium/Nutrient Mixture F-12 (DMEM/F12). All media were supplemented with 10% fetal bovine serum and antibiotics (100 U/mL penicillin and 100 ng/mL streptomycin; Invitrogen, Tokyo, Japan). Media and sera were sourced from Gibco (Thermo Fisher Scientific, USA).

### Construction of *TBRG4* shRNAs, RNA extraction, and quantitative real-time polymerase chain reaction (qRT–PCR)

We obtained the shRNAs targeting *TBRG4* from GeneChem (Shanghai, China). The detailed sequences are as follows: negative control (NC): TTCTCCGAACGTGTCACGT; shTBRG4#1 target sequence: GTTCTTCAGCCTGGTACAT; shTBRG4#2 target sequence: CCTGAATTTCACATCCAATTT. Following the transfection of lung cancer cells, total RNA was extracted using TRIzol reagent (SuperfecTRI, China) and reverse transcribed into cDNA using Promega M-MLV (Beijing, China). The resulting cDNA was analyzed by qRT–PCR (Takara, Dalian, China) on an Agilent Real-Time PCR System (Agilent, CA, USA). The primers used in this study were as follows: TBRG4: 5′-CAGCTCACCTGGTAAAGCGAT-3′ (forward) and 5′-GGGAGTAGATGCTCGTTCCTTC-3′ (reverse); GAPDH: 5′-TGACTTCAACAGCGACACCCA-3′ (forward) and 5′-CACCCTGTTGCTGTAGCCAAA-3′ (reverse).

### Cell viability and colony formation

We used the CCK-8 assay (Yeasen Biotech, Shanghai, China) to evaluate cell viability. A 100 µL cell suspension containing 10^4^ lung cancer cells was seeded into each well of a 96-well plate. After 72 h of incubation, 20 µL of CCK-8 solution was added to each well. Absorbance was measured at 450 nm using an enzyme microplate reader (TECAN, Männedorf, Switzerland). For the colony formation assay, transfected cells were seeded into six-well plates at a density of 1000 cells per well. After 7–10 days of continuous culture, colonies were stained with crystal violet, visualized under a microscope (Olympus, Shinjuku-ku, Japan), photographed, and counted.

### Apoptosis analysis

We used an Annexin V-FITC apoptosis detection kit (eBioscience, San Diego, CA, USA) to assess the apoptosis rate. Briefly, lung cancer cells were transfected with either shTBRG4 or shCtrl lentivirus. Forty-eight hours after transfection, cells in the exponential growth phase were harvested and resuspended in staining buffer at a final density of 1 × 10^6^/mL. Then, 10 µL of Annexin V-FITC was added to 100 µL of the cell suspension and incubated for 10–15 min at room temperature in the dark. After incubation, 400 µL of 1× binding buffer was added and mixed thoroughly. Flow cytometry was performed within 1 h using an FACSCalibur (Becton-Dickinson, NJ, USA) to detect apoptosis.

### Cell cycle analysis

Transfected cells in the exponential growth phase were fixed by adding pre-cooled 70% ethanol and stored at 4 ^∘^C overnight. The cells were then collected by centrifugation at 1200 rpm for 5 min and washed once with 1 mL of PBS. Next, 500 µL of PBS containing 50 µg/mL PI, 100 µg/mL RNase A, and 0.2% Triton X-100 was added to the cell pellet and incubated for 30 min at 4 ^∘^C in the dark. Cell cycle distribution was analyzed by flow cytometry, using samples containing approximately 2 × 10^5^ cells. Data were processed using ModFit software (Verity Software House, ME, USA). All experiments were performed in triplicate.

### Western blot analysis

The transfected lung cancer cells were lysed by sonication in RIPA lysis buffer (Beyotime Biotechnology, Shanghai, China). The extracted proteins were separated on a 12% SDS-PAGE and then transferred onto a PVDF membrane (Beyotime Biotechnology, Shanghai, China). Following a 2-h block with 5% nonfat milk, the membranes were incubated overnight at 4 ^∘^C with the appropriate primary antibodies. The next day, the membranes were incubated with the corresponding secondary antibodies (Beyotime Biotechnology, Shanghai, China) at room temperature for 1 h. Protein bands were visualized using an ECL detection system (Clinx, Shanghai, China). In a darkroom, equal volumes of ECL solutions A and B (Yeasen Biotech, Shanghai, China) were mixed and applied to the membrane surface. After a 1-min incubation, the PVDF membrane was placed into a film cartridge and allowed to sit for 1–10 min. The film was then developed by immersion in developer solution. Once the bands appeared, the film was immediately transferred to a fixing solution for rinsing and image capture.

### *In vivo* tumorigenesis

BALB/c mice (four weeks old) were obtained from Lingchang Biotech (Shanghai, China). Following the methodology described by Liu et al. [12], xenograft models were established by subcutaneously injecting 100 µL of NCI-H1299 cells (4 × 10^6^), stably transfected with either shTBRG4#2 or a control vector, into BALB/c mice (*n* ═ 10 per group). To generate stable shTBRG4#2 knockdown cells, NCI-H1299 cells were transfected with lentivirus expressing shTBRG4#2 using Lipofectamine 3000 (Thermo Fisher Scientific, USA), according to the manufacturer’s protocol. After 48 h, cells were selected with 2 µg/mL puromycin (Sigma-Aldrich, USA) for two weeks to ensure stable knockdown. Tumor volume was measured weekly for six weeks and calculated using the formula: Volume ═ (Length × Width^2^)/2. Additionally, tumor size was evaluated using an *in vivo* imaging system (PerkinElmer, MA, USA). D-luciferin (15 mg/mL; Qcbio Science & Technologies Co., Ltd, Shanghai, China) was administered via intraperitoneal injection (10 µL/g) 15 min prior to imaging. At the end of six weeks, mice were sacrificed and tumors were excised and weighed. All animal experiments were conducted in accordance with the Guidelines for the Care and Use of Laboratory Animals (NIH Pub. No. 85-23, revised 1996) and were approved by the Ethics Committee of the First Affiliated Hospital of Bengbu Medical College.

### Measuring immune response predictors: immune phenotype scores

The immunophenoscore (IPS) is a robust predictor of response to anti-CTLA-4 and anti-PD-1 therapies. It quantifies key determinants of tumor immunogenicity and characterizes both the intratumoral immune landscape and the cancer antigenome. The scoring system is based on a panel of immune-related genes grouped into four categories: MHC-associated molecules (MHC), checkpoints or immunomodulators (CP), effector cells (ECs), and suppressor cells (SCs). A weighted average Z-score is calculated by averaging the sample Z-scores within each category, and the overall IPS is derived by summing these weighted average Z-scores.

### Ethical statement

The animal experiments were conducted in accordance with the Guide for the Care and Use of Laboratory Animals (NIH Publication No. 85-23, revised 1996) and were approved by the Ethics Committee of the First Affiliated Hospital of Bengbu Medical University (Approval Number: 2020173).

### Statistical analysis

Student’s *t*-test was used to determine the difference after knocking down *TBRG4* expression with Microsoft Office software, and the results are displayed as the mean ± standard deviation (SD). Only a *P* value of 0.05 was regarded as statistically significant.

**Figure 1.**
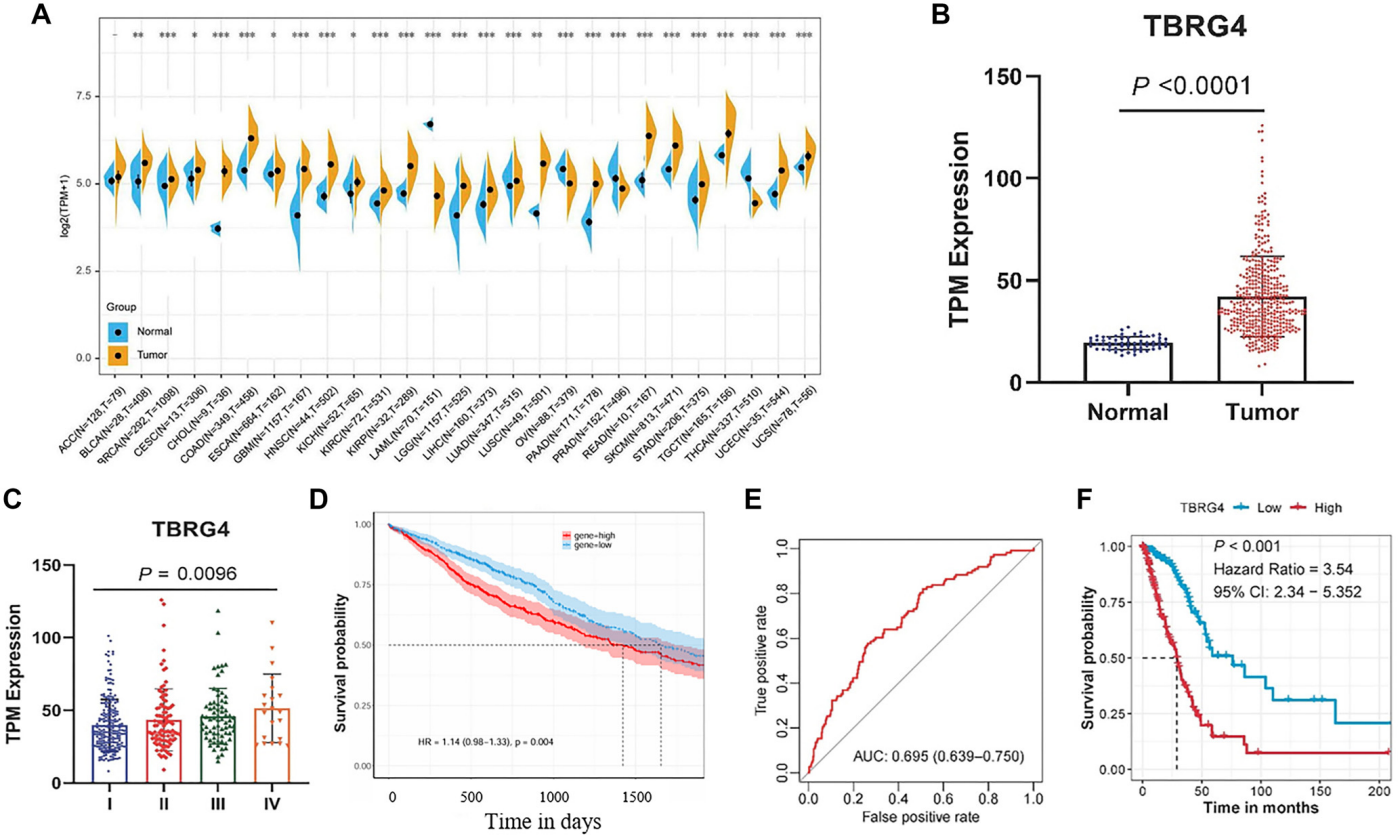
***TBRG4* expression is upregulated in multiple cancers and predicts poor prognosis in LUAD.** (A) Comparing the expression of TBRG4 between cancer tissues and normal controls at the pan-cancer level; (B) Comparing the expression difference of TBRG4 in LUAD tumor tissues and normal controls; (C) Comparing the expression differences of TBRG4 in the different clinical stages of LUAD patients; (D) Kaplan–Meier plot showing the survival difference between high TBRG4 and low TBRG4 expression subgroups; (E) ROC curve reflecting the predictive capacity of the nomogram model among LUAD patients; (F) Kaplan–Meier plot showing the survival difference between high- and low-risk subgroups of LUAD patients. LUAD: Lung adenocarcinoma; TBRG4: Transforming growth factor β regulator 4; ROC: Receiver operating characteristic.

## Results

### *TBRG4* expression is upregulated in multiple cancers and predicts poor prognosis in LUAD

**Figure 2.**
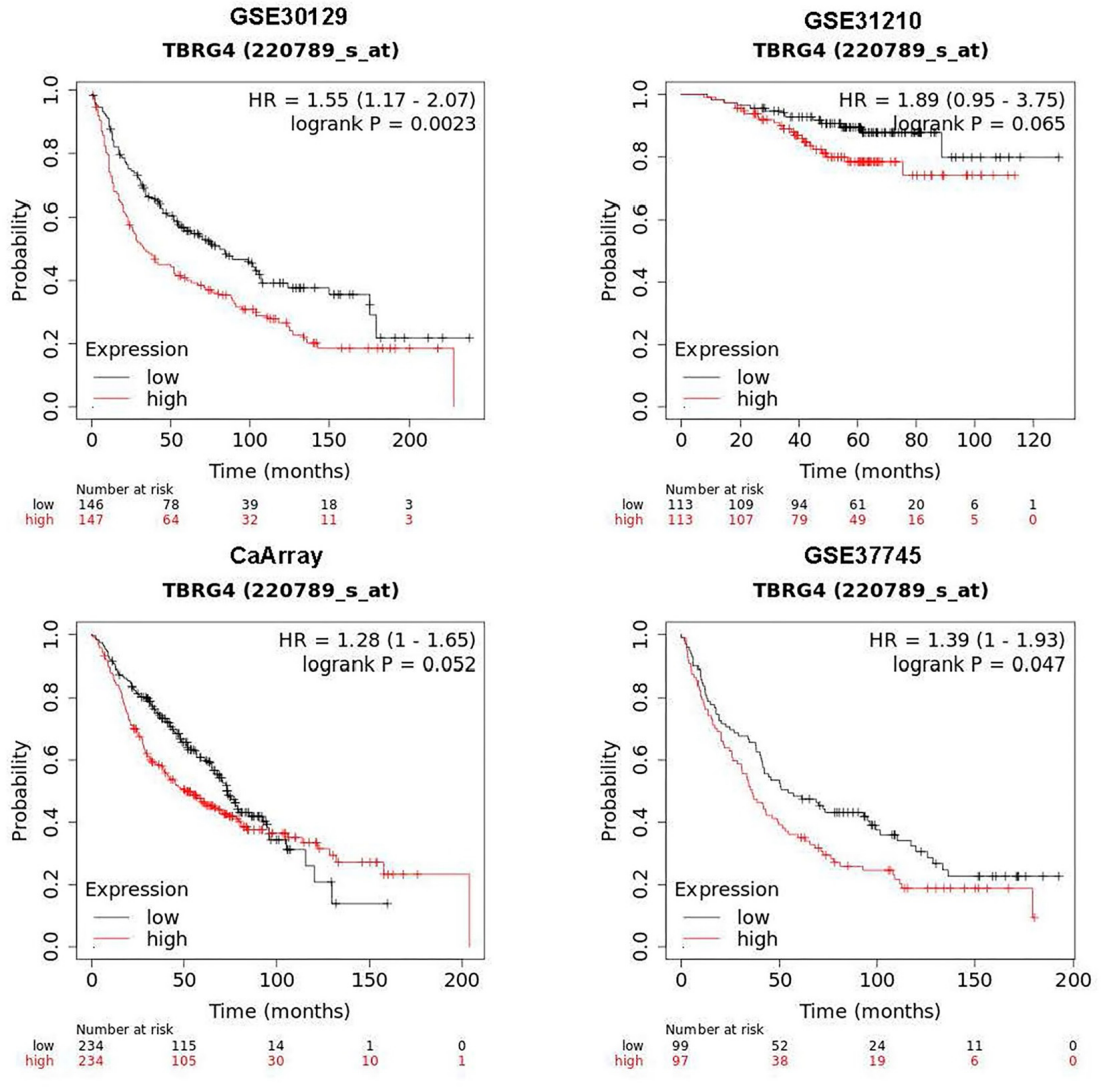
**Kaplan–Meier plot showing the survival difference between high- and low-*TBRG4* expression subgroups in GSE30129, GSE31210, CaArray, and GSE37745.** TBRG4: Transforming growth factor β regulator 4; NC: Negative control.

We examined the expression of *TBRG4* across various cancer types and found it to be significantly upregulated in tumor tissues compared to normal controls in bladder cancer, breast cancer, cervical cancer, bile duct cancer, colon cancer, esophageal cancer, glioblastoma, head and neck cancer, kidney chromophobe, kidney clear cell carcinoma, kidney papillary cell carcinoma, lower-grade glioma, liver cancer, LUAD, lung squamous cell carcinoma, pancreatic cancer, rectal cancer, melanoma, stomach cancer, testicular cancer, endometrioid cancer, and uterine carcinosarcoma. Conversely, *TBRG4* expression was downregulated in acute myeloid leukemia, ovarian cancer, prostate cancer, and thyroid cancer (Figure 1A). In our previous study, we observed a significant increase in *TBRG4* expression in lung cancer tissues compared to normal controls [9]. In the current study, we further confirmed this upregulation in tumor tissues based on expression profiles from the TCGA database (Figure 1B). Additionally, *TBRG4* expression was positively correlated with tumor stage (Figure 1C), and higher expression levels were associated with poorer prognosis in LUAD patients (HR ═ 1.72, 95% CI: 1.186–2.506, log-rank *P* ═ 0.004; Figure 1D). Multivariate Cox regression analysis confirmed that TBRG4 is an independent prognostic factor, regardless of other clinicopathological features (HR ═ 8.071, 95% CI: 1.553–41.945, *P* ═ 0.013; Table 1). We developed a nomogram incorporating TBRG4, tumor stage, and age (Table 2), and validated its prognostic performance using ROC analysis (AUC ═ 0.695, 95% CI: 0.639–0.750; Figure 1E) and Kaplan–Meier analysis (Figure 1F; log-rank *P* value < 0.01, HR ═ 3.54, 95% CI: 2.34–5.352). Furthermore, we evaluated the prognostic value of *TBRG4* in the GSE30129, GSE31210, CaArray, and GSE37745 cohorts using SurvExpress, which yielded consistent results (Figure 2). Collectively, these findings underscore the prognostic significance of the TBRG4 gene.

**Table 1:** Univariate and multivariate analyses illustrate the predictive value of *TBRG4*

**Parameters**	**HR**	**95% CI**	***P* value**
*Univariate cox regression*			
Age	1.016	0.997–1.035	0.103
Gender (Male vs Female)	0.892	0.612–1.300	0.552
Smoke (Ever-smoker vs Non-smoker)	0.900	0.497–1.627	0.726
Smoke (Smoker vs Non-smoker)	0.695	0.355–1.360	0.288
Stage (II vs I)	2.884	1.785–4.661	1.52E-05*
Stage (III vs I)	4.291	2.638–6.979	4.36E-09*
Stage (IV vs I)	3.276	1.666–6.440	0.001*
TBRG4	1.011	1.002–1.020	0.019*
*Multivariate cox regression*			
Age	1.030	1.008–1.052	0.006*
Gender (Male vs Female)	0.805	0.533–1.214	0.300
Smoke (Ever-smoker vs Non-smoker)	1.336	0.709–2.517	0.370
Smoke (Smoker vs Non-smoker)	0.838	0.406–1.728	0.632
Stage (II vs I)	3.360	2.038–5.538	2.00E-06*
Stage (III vs I)	4.064	2.474–6.678	3.13E-08*
Stage (IV vs I)	4.656	2.287–9.479	2.22E-05*
TBRG4	8.071	1.553–41.945	0.013*

*, *P* < 0.05; HR: Hazard ratio; 95% CI: 95% confidential interval; TBRG4: Transforming growth factor β regulator 4.

**Table 2:** Index for the parameters enrolled in risk score formula

	**Co-ef**	**Exp(co-ef)**	**Se(co-ef)**	**z**	***P* value**
Age	0.032	1.032	0.011	2.97	0.003
Stage (II vs I)	1.068	2.908	0.245	4.352	1.35E-05*
Stage (III vs I)	1.357	3.886	0.25	5.419	5.99E-08*
Stage (IV vs I)	1.355	3.876	0.349	3.879	1.05E-04*
TBRG4	0.503	1.653	0.186	2.703	0.007*

TBRG4: Transforming growth factor β regulator 4.

**Figure 3.**
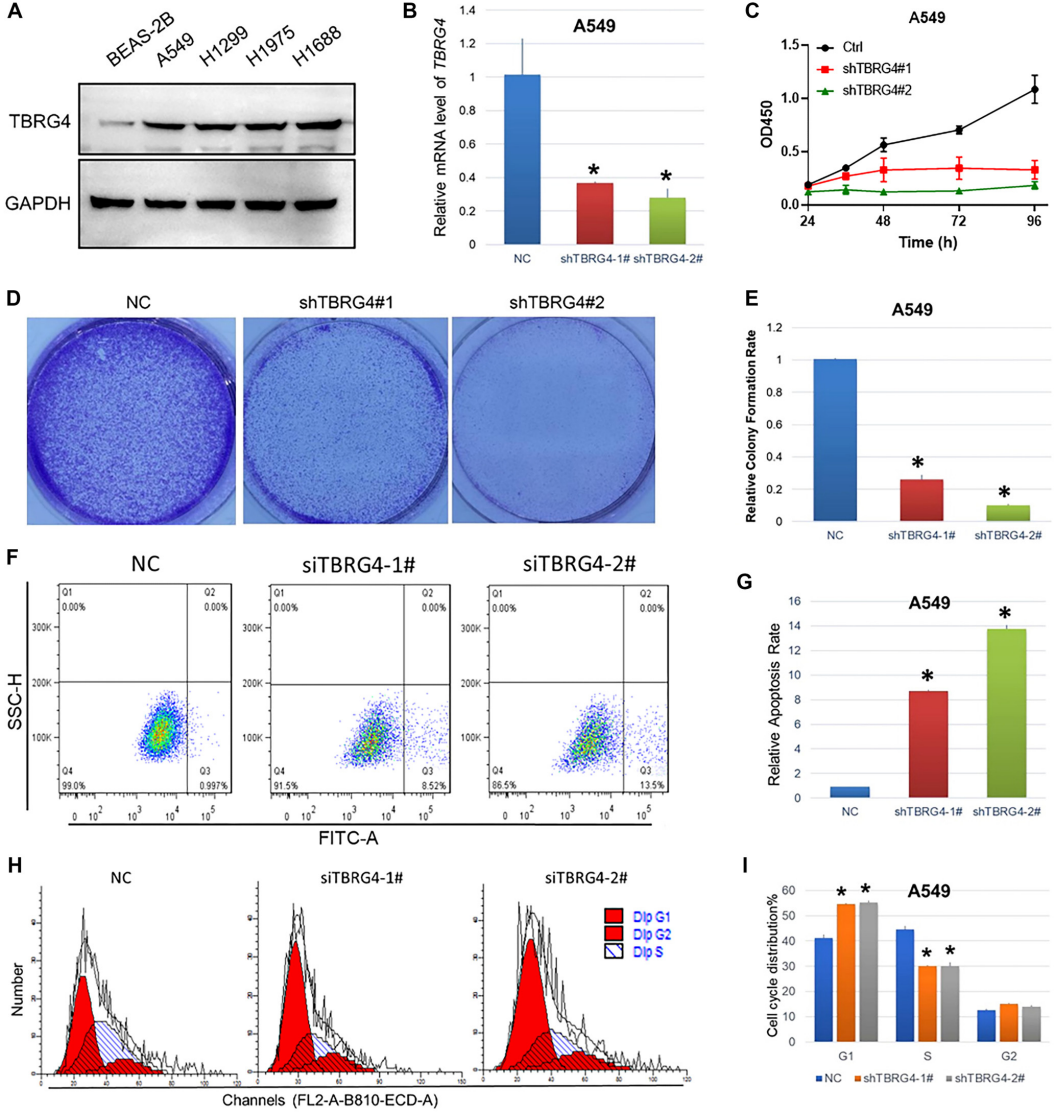
***TBRG4* knockdown inhibits A549 proliferation and induces apoptosis.** (A) The protein expression of TBRG4 difference between lung cancer cells compared with normal control; (B) The knocking down efficiencies of TBRG4 in the A549 cell line; (C) CCK-8 assay showing the proliferation difference after silencing TBRG4 expression in the A549 cell line; (D) Colony formation assay showing the colony formation capacity difference after silencing TBRG4 expression in the A549 cell line; (E) Quantification analysis of the colony formation assay; (F) Flow cytometry showing the apoptosis rate after silencing TBRG4 expression in the A549 cell line; (G) Quantification of the cell apoptosis results; (H) Flow cytometry showing the cell cycle distribution difference after silencing TBRG4 expression in the A549 cell line; (I) Quantification of the cell cycle distribution difference. *P < 0.05. TBRG4: Transforming growth factor β regulator 4; NC: Negative control.

**Figure 4.**
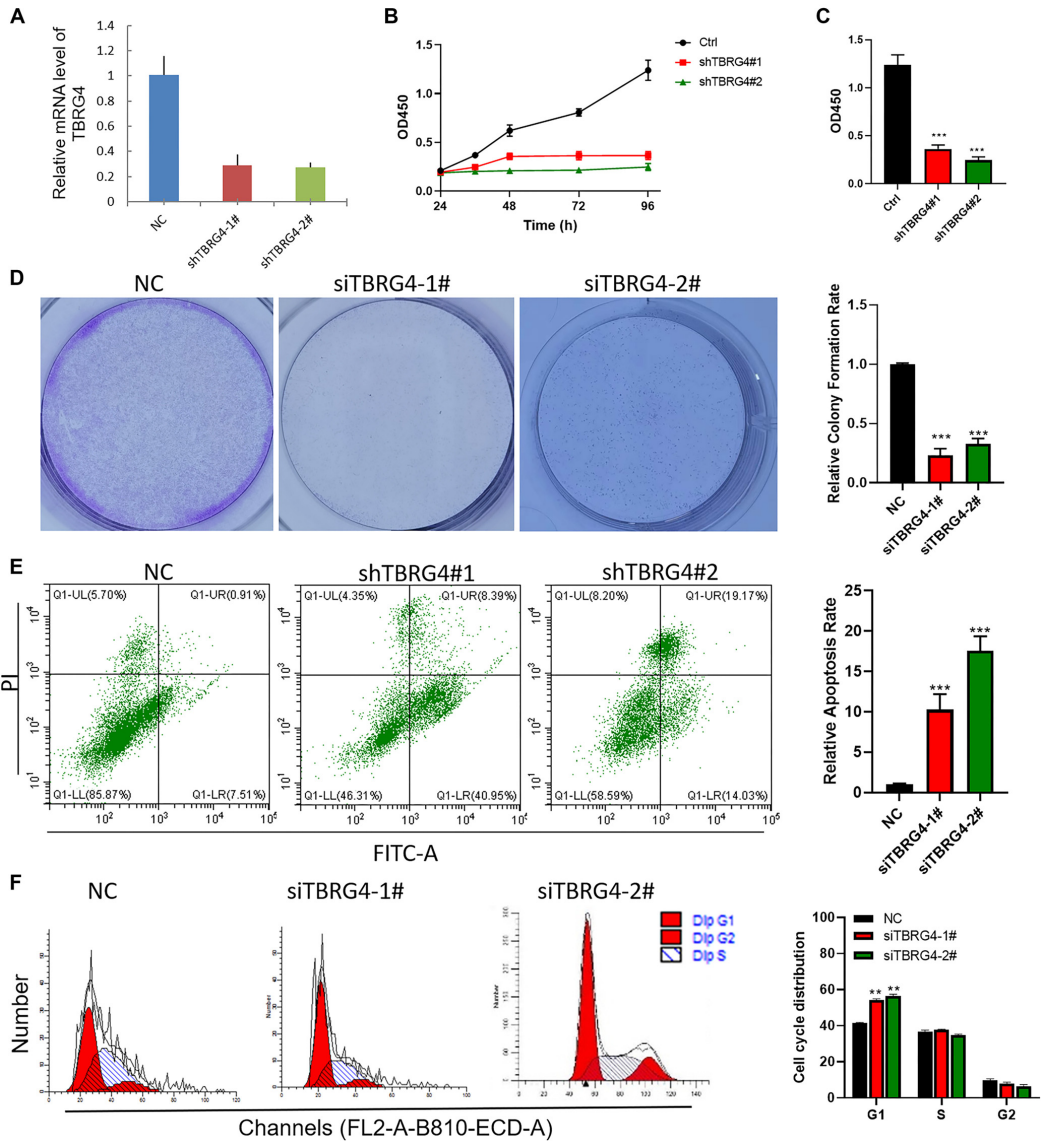
***TBRG4* knockdown inhibits H1299 proliferation and induces apoptosis.** (A) The knocking down efficiencies of TBRG4 in the H1299 cell line; (B and C) CCK-8 assay showing the proliferation difference after silencing TBRG4 expression in the H1299 cell line; (D) Colony formation assay showing the colony formation capacity difference after silencing TBRG4 expression in the H1299 cell line; (E) Flow cytometry showing the apoptosis rate after silencing TBRG4 expression in the H1299 cell line; (F) Flow cytometry showing the cell cycle distribution difference after silencing TBRG4 expression in the H1299 cell line. **P < 0.01, ***P < 0.001. TBRG4: Transforming growth factor β regulator 4; NC: Negative control.

### *TBRG4* knockdown inhibits lung cancer cell proliferation and induces apoptosis

**Figure 5.**
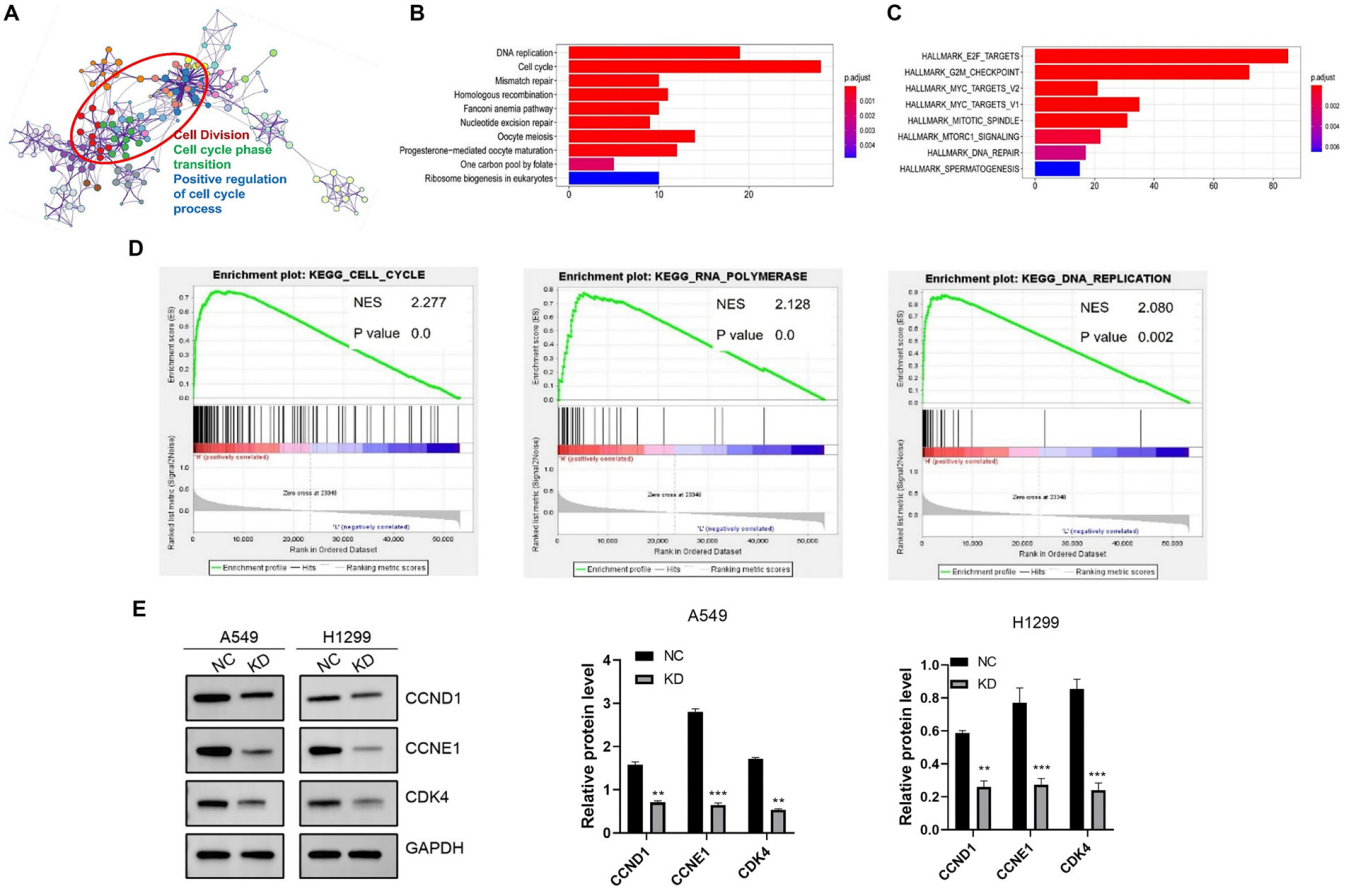
**Co-expressed genes of *TBRG4* and relevant pathway in lung cancer.** (A) Metascape KEGG; (B) Hallmark KEGG; (C) Gene Set Enrichment Analysis; (D) Analyses of the co-expressed genes of TBRG4 based on expression matrix of lung cancer obtained from TCGA database; (E) Western blot assay displaying the expression variation of critical markers in cell cycle pathway after silencing TBRG4 expression. ***P < 0.001. KEGG: Kyoto Encyclopedia of Genes and Genomes; TBRG4: Transforming growth factor β regulator 4; TCGA: The Cancer Genome Atlas.

We first assessed TBRG4 protein expression levels in four lung cancer cell lines (H1688, H1975, H1299, and A549) and one normal human lung epithelial cell line (BEAS-2B). Our results indicated that *TBRG4* was expressed at significantly higher levels in cancer cells compared to the normal control (Figures 3A and S1A). Based on these findings, we randomly selected two lung cancer cell lines, H1299 and A549, for further biological experiments. Following *TBRG4* knockdown, we observed a significant reduction in cell proliferation and colony formation in both H1299 and A549 cells (Figures 3B–3E, S1B and S1C, and 4A–4D). To determine whether *TBRG4* knockdown selectively affects cancer cells, we also assessed its impact on the normal lung epithelial cell line BEAS-2B. The results showed only a slight, non-significant change in BEAS-2B cell growth, suggesting that *TBRG4* knockdown has a limited effect on normal cells. This supports the idea that *TBRG4* plays a more selective role in cancer cells (Figure S1D and S1E). In addition, *TBRG4* knockdown significantly increased the apoptosis rate in both cancer cell lines (Figures 3F–3G and 5E). We also analyzed cell cycle distribution by flow cytometry. The results revealed a marked increase in the G1 phase and a corresponding decrease in the S and G2 phases in H1299 and A549 cells following *TBRG4* knockdown (Figures 3H–3I and 4F). These findings suggest that *TBRG4* is critically involved in regulating proliferation, apoptosis, and cell cycle progression in lung cancer cells. *TBRG4* may represent a potential therapeutic target, and interventions aimed at inhibiting its function could help suppress lung cancer growth and progression.

### Exploring the underlying mechanisms

**Figure 6.**
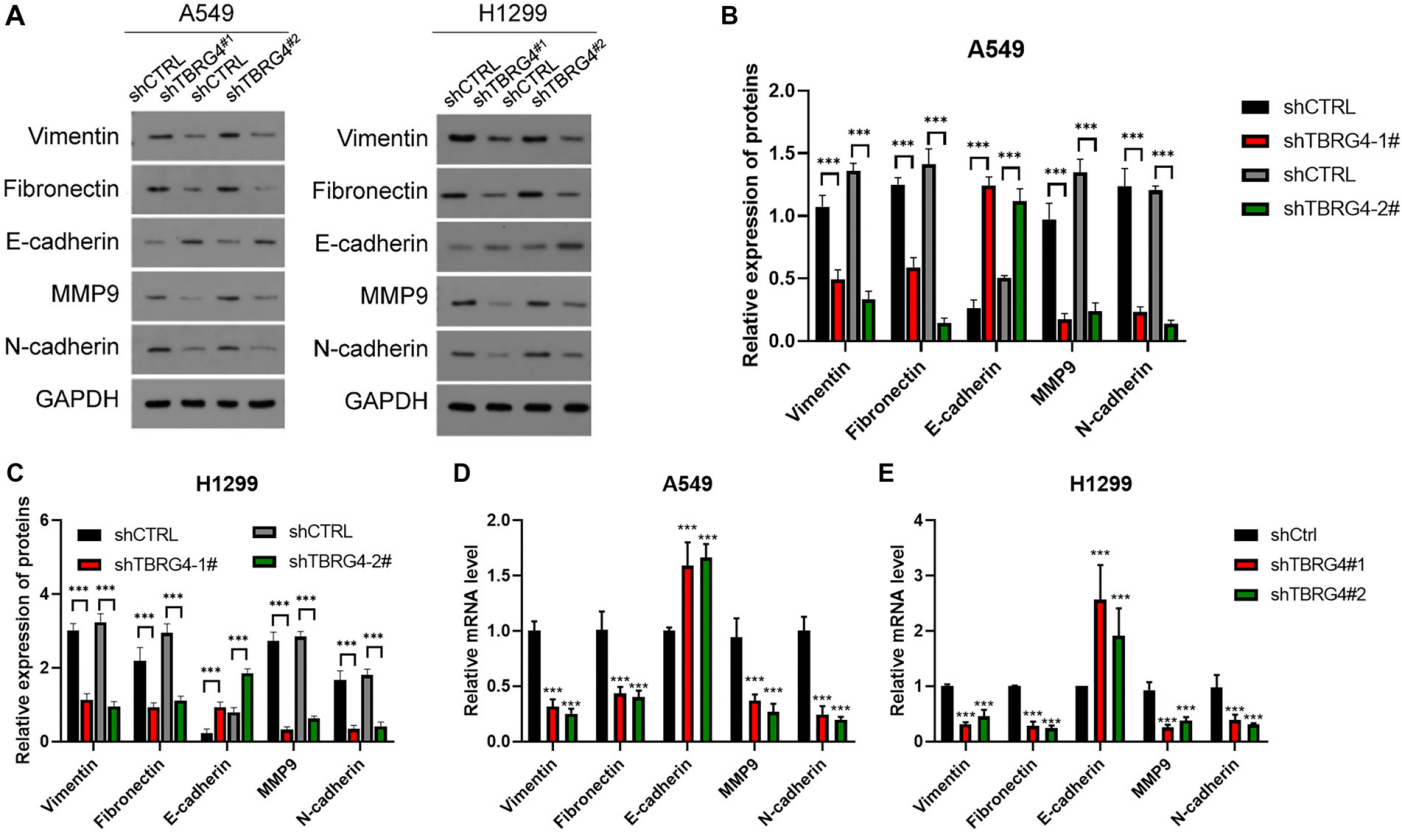
**Silencing the expression of *TBRG4* suppressed the EMT pathway in both A549 and H1299 cell lines.** (A–C) Western blot and (D and E) qPCR were used to detect the expression levels of EMT markers, including vimentin, fibronectin, MMP9, and N-cadherin, after silencing TBRG4, ***P < 0.001. TBRG4: Transforming growth factor β regulator 4.

To investigate the underlying mechanisms, we analyzed genes coexpressed with *TBRG4* using the TCGA-PRAD cohort, identifying those with a Pearson correlation coefficient greater than 0.4. KEGG, Hallmark, and Metascape pathway analyses revealed that these coexpressed genes were predominantly enriched in cell cycle-related processes, including cell division, phase transition, and positive regulation of the cell cycle (Figure 5A–5D), consistent with our previous findings. Building on this, we performed a western blot assay to determine whether *TBRG4* knockdown affects the cell cycle pathway. Our results showed that silencing *TBRG4* reduced the expression of key cell cycle markers, including CCND1, CDK4, and CCNE1 (Figure 5E). We also examined the effect of *TBRG4* knockdown on the EMT pathway. Western blot analysis revealed that silencing *TBRG4* significantly decreased the expression of mesenchymal markers, such as Vimentin, Fibronectin, MMP9, and N-cadherin (Figure 6A–6C). In contrast, it markedly increased the expression of the epithelial marker E-cadherin. These results were further validated by qPCR (Figure 6D and 6E). Together, these findings suggest that *TBRG4* plays a critical role in promoting EMT, as indicated by the downregulation of mesenchymal markers and the upregulation of epithelial markers. This shift in protein expression patterns implies that *TBRG4* may regulate the balance between epithelial and mesenchymal phenotypes, potentially influencing tumor invasiveness and metastatic potential.

### *TBRG4* knockdown inhibits tumor growth in xenograft lung cancer models

**Figure 7.**
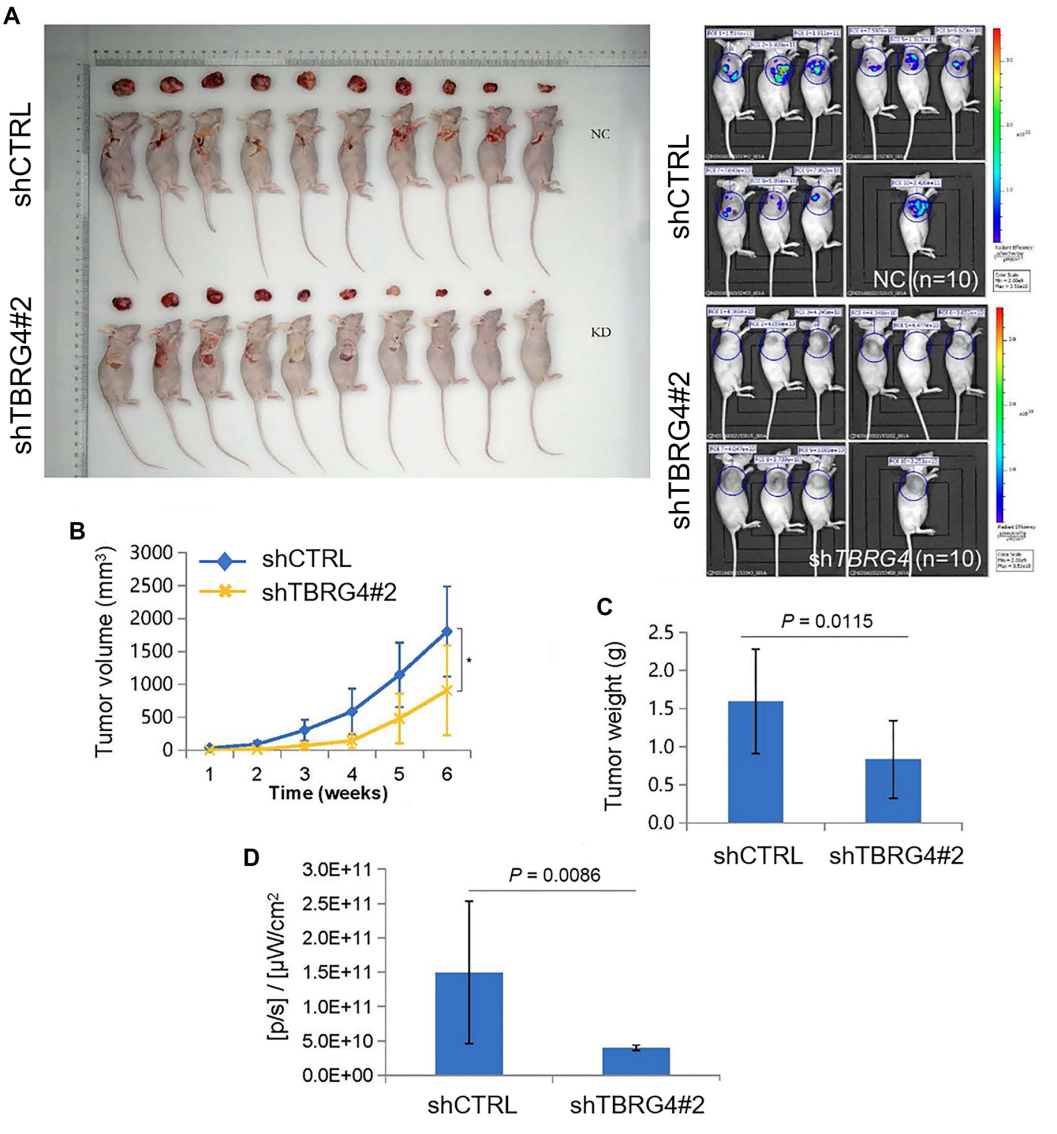
***In vivo* analysis of tumor growth in xenograft models.** (A and B) Mice were subcutaneously injected with H1299 cells transfected with shTBRG4 or control shRNA. Tumor volume was measured weekly starting from day seven post-injection. The results show that tumors in the shTBRG4 group were significantly smaller than those in the negative control group at 42 days post-injection (*n* ═ 10 mice per group, *P* < 0.05). (C) At the end of the experimental period, tumors were excised and weighed. Tumor weights in the shTBRG4 subgroup were significantly lower compared to the negative control group (mean ± SD, *n* ═ 10, *P* < 0.01). (D) *In vivo* imaging of tumors demonstrated reduced bioluminescence in the shTBRG4 group, indicating lower tumor activity. Statistical analyses were performed using Student’s *t*-test. *P < 0.05. TBRG4: Transforming growth factor β regulator 4; SD: Standard deviation.

Next, we established xenograft lung tumor models using BALB/c nude mice to evaluate the impact of *TBRG4* knockdown on tumor growth *in vivo*. Between the two knockdown constructs, shTBRG4#2 demonstrated superior knockdown efficiency and functional impact compared to shTBRG4#1, and was therefore selected for subsequent *in vivo* experiments. Specifically, H1299 lung cancer cells transfected with either shTBRG4#2 or control shRNA were injected subcutaneously into the flanks of the mice. Tumor development was monitored weekly, with volume measurements beginning on day seven post-injection. As shown in Figure 7A and 7B, mice injected with *TBRG4* knockdown cells (shTBRG4#2 group) exhibited significantly reduced tumor growth compared to controls. Tumors in the shTBRG4#2 group were visibly smaller, indicating a marked suppression of tumor progression. After 42 days, the mice were sacrificed and tumor weights were recorded. As illustrated in Figure 7C, the average tumor weight in the shTBRG4#2 group was significantly lower than in the control group, confirming a strong inhibitory effect of *TBRG4* knockdown on tumor growth. This reduction in tumor burden was further supported by *in vivo* bioluminescence imaging (Figure 7D), which showed consistently diminished tumor activity in the shTBRG4#2 group throughout the experimental period. Taken together, these results indicate that silencing *TBRG4* effectively inhibits tumor growth *in vivo*. The consistent findings across both physical measurements and imaging data underscore the potential of *TBRG4* as a therapeutic target for suppressing tumor progression.

### Potential role of *TBRG4* in response to immunotherapy in lung cancer

**Figure 8.**
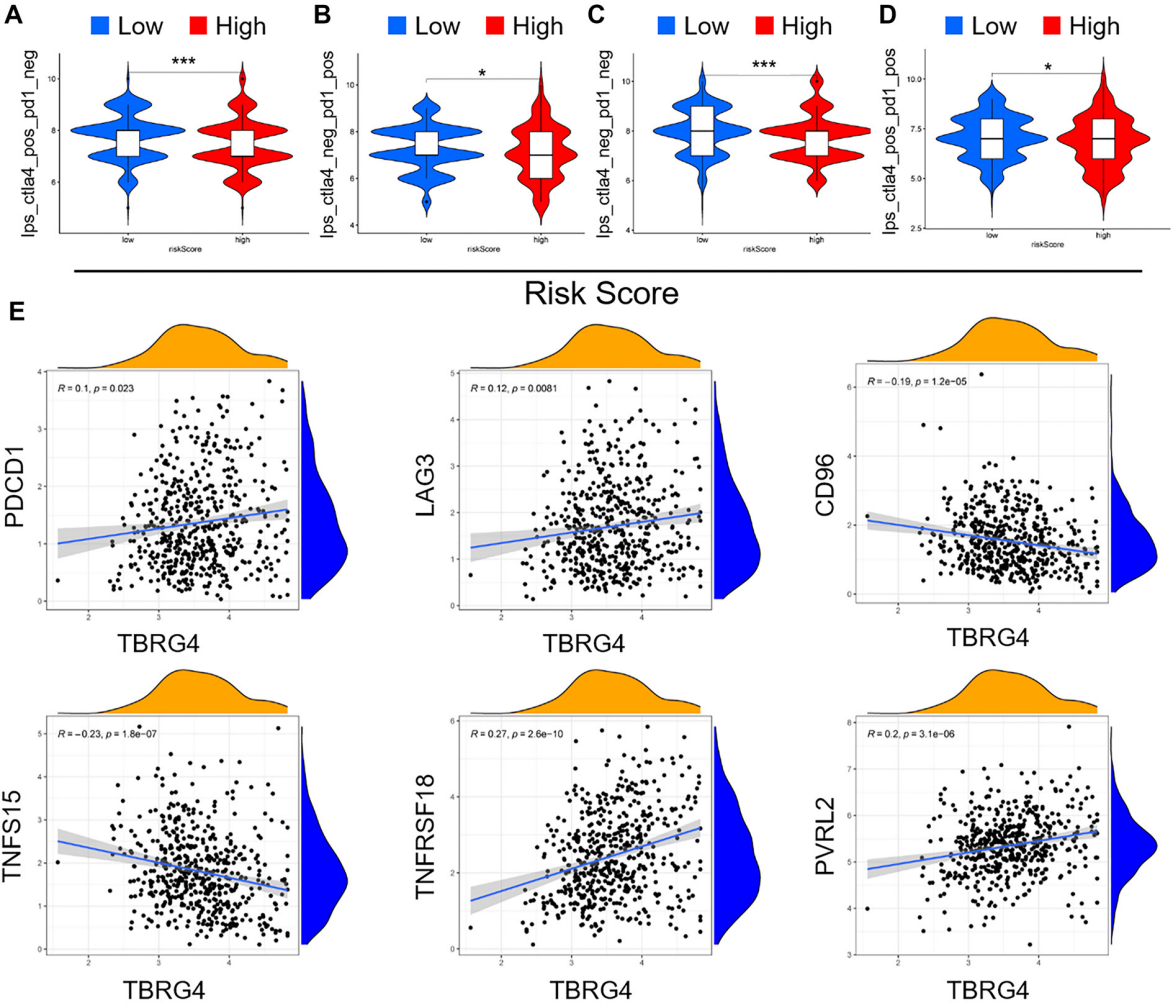
**The potential role of *TBRG4* in immunotherapy response in lung cancer.** (A–D) Violin plots comparing the expression of various immune checkpoint indicators between low-risk (blue) and high-risk (red) groups. (A) ips_ctla4_pos_pd1_neg indicates response to anti-CTLA-4 and no response to PD-1; (B) ips_ctla4_neg_pd1_pos indicates no response to anti-CTLA-4 and response to PD-1; (C) ips_ ctla4_neg_pd1_neg indicates no response to anti-CTLA-4 and anti-PD-1 antibodies; (D) ips_ctla4_pos_pd1_pos indicates response to both anti-CTLA-4 and anti-PD-1 antibodies. *P < 0.05, ***P < 0.001; (E) Scatter plots illustrating the correlation between TBRG4 expression and various immune checkpoint molecules: PDCD1, LAG3, CD96, TNFSF15, TNFRSF18, and PVRL2. Each plot includes the correlation coefficient (R) and the *P* value, indicating the strength and significance of the correlation. TBRG4: Transforming growth factor β regulator 4.

To further explore the relationship between *TBRG4* expression and immunotherapy responses in lung cancer, we evaluated whether *TBRG4* could serve as a predictor of patient outcomes following ICI therapy. Alongside established biomarkers, such as TML, PD-L1 expression, and MSI, emerging indicators like the IPS are gaining attention for their utility in assessing immune responses. Our analysis revealed significantly higher IPS values in the low *TBRG4* expression group among PD-1–negative and CTLA-4–positive patients, whereas the high *TBRG4* expression group showed markedly lower IPS values (Figure 8A–8D). These findings suggest that reduced *TBRG4* expression may be linked to enhanced immune responsiveness or a more favorable prognosis. Given the pivotal role of immune checkpoints in determining immunotherapy efficacy, we also examined the relationship between *TBRG4* expression and six key immune checkpoint genes. As shown in Figure 8E, TBRG4 expression positively correlated with PDCD1 (PD-1), LAG3, TNFRSF18, and PVRL2 (*P* < 0.05, *R* > 0.1), indicating that higher *TBRG4* expression may enhance immune checkpoint signaling—potentially suppressing T cell activation and contributing to immune evasion. Conversely, TBRG4 showed a negative correlation with CD96 and TNFSF15 (*P* < 0.05, R < −0.1), suggesting it may influence pathways that inhibit T cell function, thereby fostering a more immunosuppressive tumor microenvironment. These results underscore the regulatory role of *TBRG4* in immune responses and its potential influence on immunotherapy effectiveness via modulation of immune checkpoint activity.

## Discussion

Lung cancer remains the leading cause of cancer-related mortality worldwide, and despite therapeutic advancements, its prognosis is still poor. This study investigates the regulatory mechanisms underlying LUAD progression and identifies *TBRG4* as a pivotal oncogenic modulator and prognostic biomarker. Our findings show that *TBRG4* is aberrantly overexpressed in multiple malignancies, including LUAD, with elevated expression significantly associated with advanced TNM stages and reduced overall survival. Mechanistically, *TBRG4* promotes tumor invasiveness through activation of the PI3K/AKT pathway and contributes to chemotherapy resistance by enhancing DNA repair capacity. These results position *TBRG4* as a dual-functional biomarker with independent prognostic value and therapeutic potential, offering novel insights for early diagnosis and targeted intervention in LUAD. *TBRG4* has previously been implicated in multiple myeloma [13], oral squamous cell carcinoma [14], and breast cancer [15], where it functions as an oncogene. Our findings further establish *TBRG4* as a critical player in LUAD, with its depletion significantly impairing cell viability and inducing apoptosis. We hypothesize that this growth suppression is at least partly due to increased apoptotic activity. In addition to its role in cell cycle regulation, pathway analysis of co-expressed genes links *TBRG4* to EMT, a key process in cancer metastasis. Our experimental assays confirmed that *TBRG4* depletion reduces EMT marker expression and suppresses tumor growth *in vivo*. These results highlight TBRG4’s dual role in lung cancer progression—regulating both the cell cycle and EMT—and underscore its potential as a prognostic biomarker and therapeutic target. Beyond its impact on LUAD progression, *TBRG4* may also modulate the tumor immune microenvironment. Emerging evidence suggests that genes involved in cell cycle regulation often intersect with immune-related pathways, influencing tumor immunogenicity and response to ICIs [16, 17]. Our analysis revealed a correlation between lower *TBRG4* expression and increased IPS in patients treated with ICIs, suggesting that *TBRG4* may serve as a predictive marker for immunotherapy responsiveness. However, our current analysis is limited to retrospective bioinformatics data from public cohorts, which may not fully capture patient heterogeneity or confounding clinical variables (e.g., comorbidities, prior treatments). Given the growing importance of immunotherapy in lung cancer treatment, further investigation into *TBRG4*’s role in immune checkpoint regulation and its interaction with the tumor immune microenvironment could uncover novel therapeutic strategies. Moreover, the link between *TBRG4* and mitochondrial function adds another layer of complexity to understanding its role in cancer. Mitochondrial dysfunction is increasingly recognized as a hallmark of cancer, contributing to altered metabolism, resistance to apoptosis, and therapeutic evasion [18]. Prior studies have proposed *TBRG4* as a mitochondrial-associated gene involved in RNA homeostasis [19, 20]. Although our study did not directly examine *TBRG4*’s role in metabolism, the observed reduction in tumor growth following *TBRG4* depletion raises the possibility of its involvement in metabolic reprogramming. Whether *TBRG4* intersects with mitochondrial metabolism to fuel tumor progression remains an open question. Metabolic profiling of *TBRG4*-depleted cells may help elucidate this potential link, bridging its molecular functions with cancer cell adaptability.

While our study provides compelling evidence for *TBRG4*’s oncogenic role, several limitations warrant consideration. First, the functional experiments were primarily conducted in two LUAD cell lines (A549 and H1299), which may not fully capture the genetic heterogeneity of clinical LUAD subtypes. Future research should validate these findings using patient-derived organoids or additional models, such as KRAS-mutant or EGFR-mutant cell lines, to ensure broader applicability. Second, although we observed TBRG4’s association with EMT and the PI3K/AKT pathway, the precise molecular mechanisms remain unclear—specifically, whether *TBRG4* directly regulates EMT transcription factors (e.g., SNAIL, TWIST) or physically interacts with PI3K subunits. Proteomic or ChIP-seq analyses will be necessary to identify *TBRG4*’s binding partners and downstream effectors. Third, our *in vivo* experiments relied on subcutaneous xenograft models, which do not fully replicate the native lung microenvironment or include an intact immune system. Future studies using orthotopic lung cancer models or syngeneic immunocompetent systems would better reflect *TBRG4*’s role in tumor–stroma interactions and responses to immunotherapy. Lastly, the clinical relevance of *TBRG4* as a predictive biomarker for chemotherapy or immunotherapy remains to be validated in prospective cohorts with standardized treatment protocols.

## Conclusion

In conclusion, our findings highlight the critical role of *TBRG4* in lung cancer prognosis and tumorigenesis. Suppressing *TBRG4* expression appears to inhibit lung cancer progression by modulating the cell cycle and EMT pathways, while also potentially affecting immune responses and mitochondrial function. Nonetheless, further research is necessary to fully elucidate the molecular mechanisms underlying *TBRG4*’s role in lung cancer and to assess its viability as a therapeutic target, particularly in the contexts of immunotherapy and cancer metabolism.

## Supplemental data

**Figure S1.**
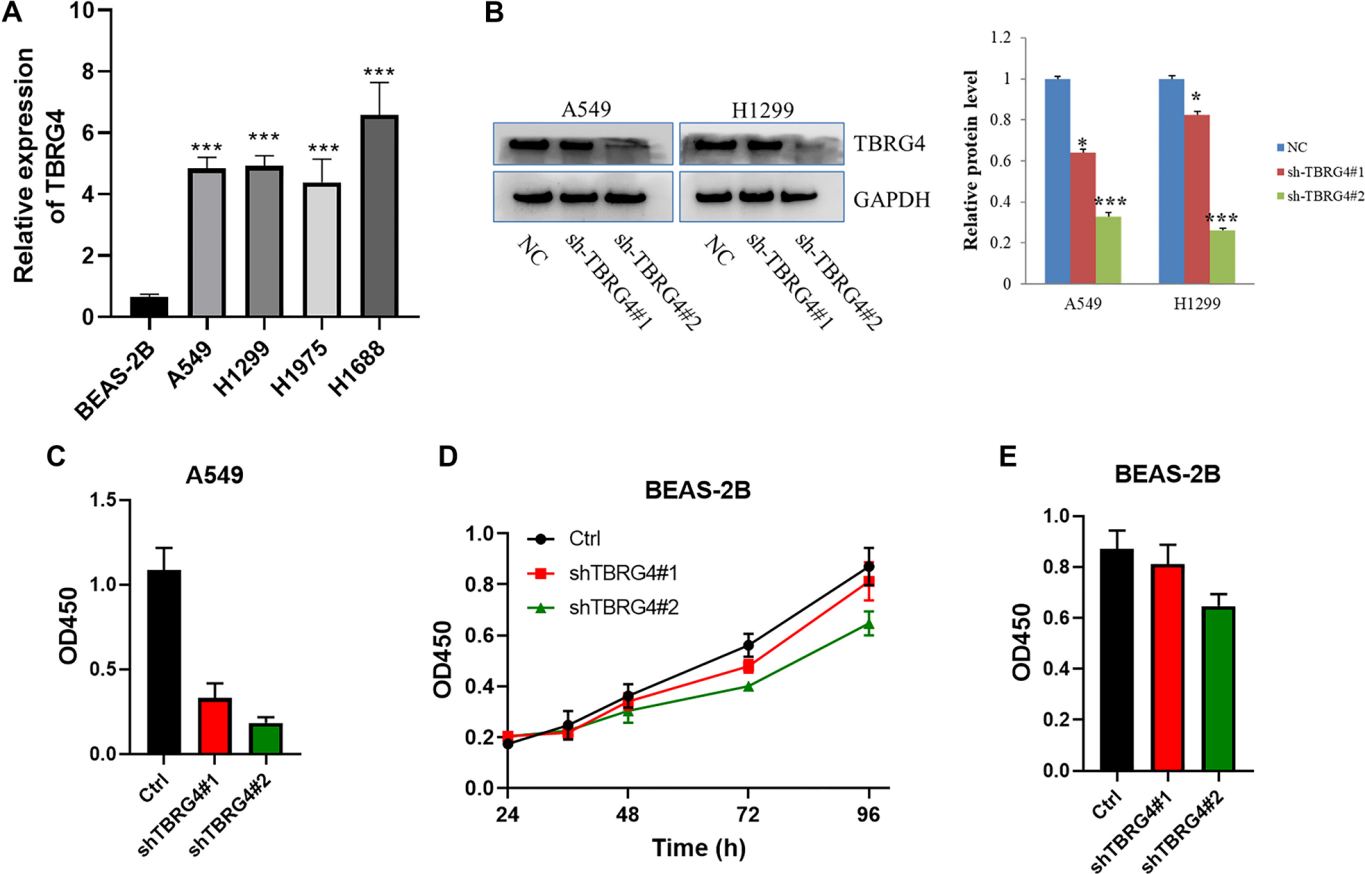
(A) The protein expression of TBRG4 difference between lung cancer cells compared with normal control; (B) Western blot analysis of TBRG4 expression in A549 and H1299 cells transfected with shTBRG4#1 or shTBRG4#2; (C) CCK-8 assay showing the proliferation difference after silencing TBRG4 expression in the A549 cell line; (D and E) CCK-8 assay showing the proliferation difference after silencing TBRG4 expression in the BEAS-2B cell line.

## Data Availability

The data used to support the findings of this study are available from the corresponding author upon request
